# The complete mitochondrial genome of a new deep-sea hagfish *Eptatretus* sp. Nan-Hai (Myxinidae: Eptatretus) from the South China Sea

**DOI:** 10.1080/23802359.2019.1711220

**Published:** 2020-01-14

**Authors:** Chun-Ang Lian, Jun-Yuan Li, Fang-Chao Zhu, Jun Li, Li-Sheng He

**Affiliations:** a Institute of Deep-sea Science and Engineering, Chinese Academy of Sciences, Sanya, China;; b College of Earth Sciences, University of Chinese Academy of Sciences, Beijing, China

**Keywords:** Mitochondrial genome, deep sea, hagfish

## Abstract

In this study, the complete mitochondrial DNA sequence of a hagfish *Eptatretus* sp. Nan-Hai from a depth of 1000 m is presented. The complete sequence was determined using next-generation sequencing and long PCRs. The mitochondrial genome of *Eptatretus* sp. Nan-Hai is 17,538 bps in size and composed of 13 protein-coding genes, two ribosomal RNA genes, 22 transfer RNA genes, and one control region (D-loop). The base composition of mitochondrial genome is biased toward A + T content, at 67.21%, with GC skew of −0.35 and AT skew of −0.03. A phylogenetic tree revealed that within the genus *Eptatretus*, *Eptatretus* sp. Nan-Hai is closely related to *Eptatretus atami*.

Hagfishes, a cosmopolitan group of craniate chordates, are considered to be the most ancient of the jawless fishes, which have attracted attention owing to their position at a crucial point in the evolutionary transition to a truly vertebrate (Ota et al. 2007). Recently, it was shown that hagfishes have the ability to find and take advantage of falling carrion faster than most other benthic scavengers, and a strategy of active predation is also adopted by hagfishes to maintain large biomasses (Tamburri and Barry 1999; Auster and Barber 2006; McLeod and Wing 2007; Zintzen et al. 2011). Thus, hagfish displays a pivotal biological status in benthic ecosystems. In this study, we sequenced the complete mitochondrial genome of *Eptatretus* sp. Nan-Hai to further analysis the genome features and phylogenetic relationships of *Eptatretus*. The present study contributes new data which could be used for both genomic and evolutionary research on hagfishes.

The specimen was trapped from the South China Sea (110.4420 E, 17.5029 N) at depth of 1000 m in June 2018, with a lander named Feng–Huang and deposited in the specimen room of the protein research lab of the Institute of Deep-sea Science and Engineering, Chinese Academy of Sciences (accession no. Nanhai-20180613FH16-HG1). Total genomic DNA was prepared from muscle tissue using an E.Z.N.A.^®^ Tissue DNA Kit (OMEGA, China), according to the manufacturer’s instructions. A paired-end library was prepared using TruSeq DNA Sample Prep Kit (Illumina, USA) and sequenced using Illumina HiSeq 2000 (2 × 150 bp paired-end reads) (Illumina, USA). After obtaining raw data, adapters and low-quality bases (<15) were removed from raw data using Trimmomatic 0.36 (Bolger et al. 2014), followed by assembled using SPAdes 3.11.0 (Bankevich et al. 2012) with defaulted parameters. The mitochondrial genome (GenBank Accession No.: MN737510) was checked again using long PCR reactions with custom primers. The mitogenome was annotated using MITOS (Bernt et al. 2013) and checked using NCBI online tools. Transfer RNA genes were further confirmed using tRNAscan-SE 1.21 (Lowe and Eddy 1997) programs.

The mitochondrial genome of *Eptatretus* sp. Nan-Hai is 17,538 bp in length. The size of *Eptatretus* sp. Nan-Hai mitogenome is similar to that of *Eptatretus burgeri* (17,168 bp). The mitogenome of *Eptatretus atami* is slight shorter with 15,298 bp, but it is not complete. The mitogenome of *Eptatretus* sp. Nan-Hai contains 13 protein-coding genes (PCGs), 22 transfer RNA genes (tRNAs), two ribosomal RNA (12S rRNA and 16S rRNA) genes, and a non-coding control region (D-loop). Except for one PCG (*nad6*) and eight tRNA genes (*tRNA^Glu^*, *tRNA^Pro^*, *tRNA^Ser^*, *tRNA^Tyr^*, *tRNA^Cys^*, *tRNA^Asn^*, *tRNA^Ala^*, *tRNA^Gln^*) coded on the light (L)-strand, all the other genes were coded on the heavy (H)-strand. This coding phenomenon of mitogenome is consistent with that of *E. burgeri.* The overall base composition of *Eptatretus* sp. Nan-Hai mitogenome is estimated to be 32.68% A, 34.53% T, 10.58% G and 22.21% C, and biased toward A + T content at 67.21%, similar to the mitochondrial genomes of *E. atami* and *E. burgeri*. The AT and GC skew of the *Eptatretus* sp. Nan-Hai mitochondrial genome are −0.03 and −0.35, respectively. Here, we also compared three mitochondrial genomes of hagfishes to analyze gene arrangements. The order of the genes on the mitochondrial genomes of *Eptatretus* sp. Nan-Hai, *E. atami* and *E. burgeri* is identical.

A maximum-likelihood phylogenetic tree of *Eptatretus* sp. Nan-Hai and 13 other species affiliated with Cyclostomata was constructed with the concatenated 13 PCGs and two rRNAs using raxmlGUI (Silvestro and Michalak 2012). The result shows that *Eptatretus* sp. Nan-Hai is closely related to *E. burgeri* (Figure 1)*.*

## Figures and Tables

**Figure 1.**
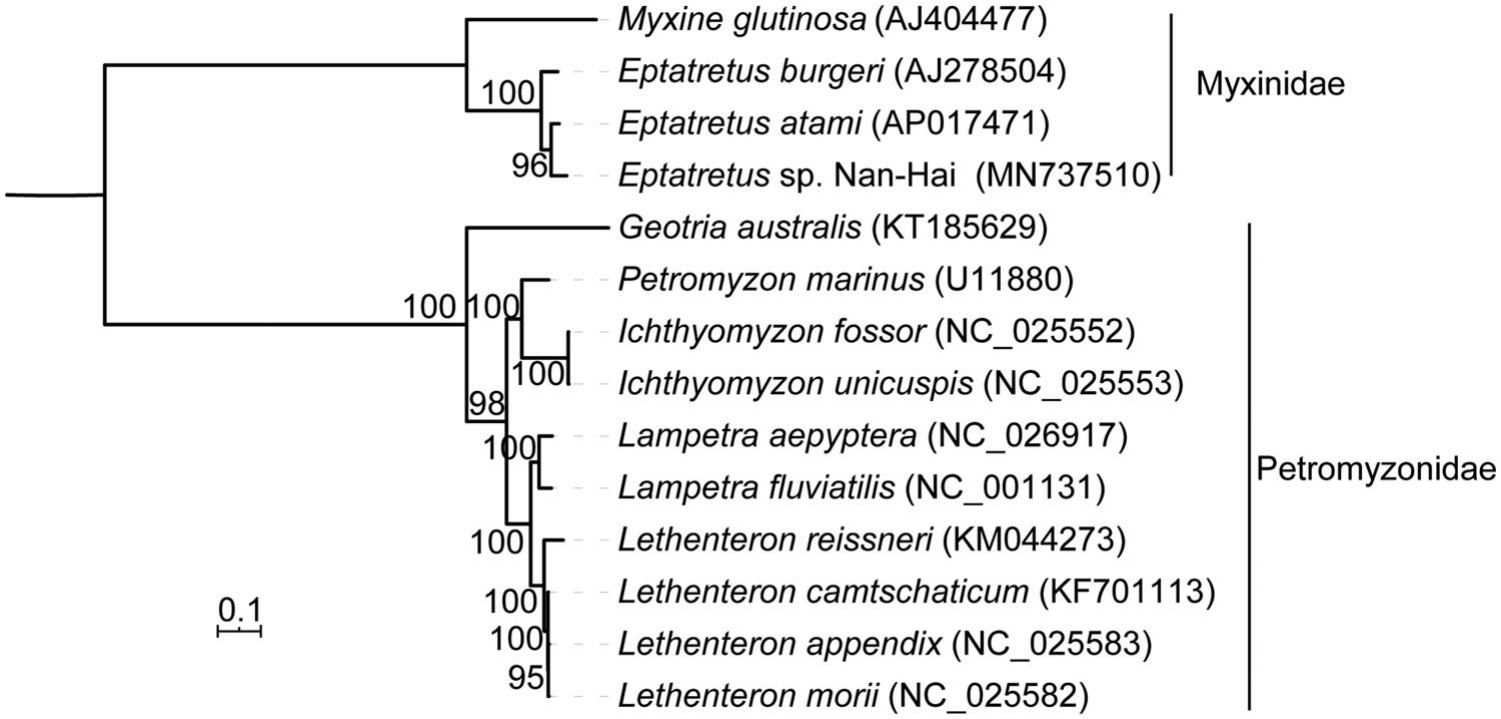
Maximum-likelihood phylogenetic tree was inferred from the concatenated nucleotide sequences of 13 PCGs and two rRNAs.
